# Parentage of Overlapping Offspring of an Arboreal-Breeding Frog with No Nest Defense: Implications for Nest Site Selection and Reproductive Strategy

**DOI:** 10.1371/journal.pone.0123221

**Published:** 2015-04-02

**Authors:** Wan-Ping Tung, Yi-Huey Chen, Wei-Chun Cheng, Ming-Feng Chuang, Wan-Tso Hsu, Yeong-Choy Kam, Richard M. Lehtinen

**Affiliations:** 1 Department of Life Science, Tunghai University, Taichung 407, Taiwan; 2 Department of Life Science, Chinese Culture University, Taipei, Taiwan; 3 Department of Biology, The College of Wooster, Wooster, Ohio 44691, United States of America; Trier University, GERMANY

## Abstract

Overlapping offspring occurs when eggs are laid in a nest containing offspring from earlier reproduction. Earlier studies showed that the parentage is not always obvious due to difficulties in field observation and/or alternative breeding tactics. To unveil the parentage between overlapping offspring and parents is critical in understanding oviposition site selection and the reproductive strategies of parents. Amplectant pairs of an arboreal-breeding frog, *Kurixalus eiffingeri*, lay eggs in tadpole-occupied nests where offspring of different life stages (embryos and tadpoles) coexist. We used five microsatellite DNA markers to assess the parentage between parents and overlapping offspring. We also tested the hypothesis that the male or female frog would breed in the same breeding site because of the scarcity of nest sites. Results showed varied parentage patterns, which may differ from the phenomenon of overlapping egg clutches reported earlier. Parentage analyses showed that only 58 and 25% of the tadpole-occupied stumps were reused by the same male and female respectively, partially confirming our prediction. Re-nesting by the same individual was more common in males than females, which is most likely related to the cost of tadpole feeding and/or feeding schemes of females. On the other hand, results of parentage analyses showed that about 42 and 75% of male and female respectively bred in tadpole-occupied stumps where tadpoles were genetically unrelated. Results of a nest-choice experiment revealed that 40% of frogs chose tadpole-occupied bamboo cups when we presented identical stumps, without or with tadpoles, suggesting that the habitat saturation hypothesis does not fully explain why frogs used the tadpole-occupied stumps. Several possible benefits of overlapping offspring with different life stages were proposed. Our study highlights the importance of integrating molecular data with field observations to better understand the reproductive biology and nest site selection of anuran amphibians.

## Introduction

Overlapping offspring is an interesting phenomenon that occurs more commonly than originally thought, but the patterns, causes, and ecological and evolutionary consequences are not fully understood. When sites are reused for reproduction, the sites may be empty (if previous offspring have left) or may contain offspring from earlier reproduction. If offspring from previous reproduction remain, this results in two overlapping cohorts. Overlapping offspring are commonly found in many oviparous animals such as insects [1, 2], fishes [3, 4], amphibians [5–8] and birds [9–12]. Often, it is assumed that the overlapping offspring are produced by the same parents; however, the parentage of offspring is not always obvious due to difficulties in field observation and/or alternative breeding tactics [13–15]. For example, when an intruding male fish takes over an egg-filled nest, it attends the eggs but also breeds with other females, resulting in overlapping offspring with multiple parentage [13, 16]. Similarly, in a salamander (*Hemidactylium scutatum*), joint nesting, where several females lay eggs at a site, can also result in complex parentage [17–19].

The parentage between overlapping offspring and nest users could lead to differences in reproductive strategy and nest site selection. When an adult reuses a nest occupied by a conspecific, it may contain genetically related or unrelated offspring. The former could be a case of nest-site fidelity [20, 21] whereas the latter could be a case of oviposition site selection [22–25]. Breeding site fidelity can be viewed as beneficial because the familiarity with an area could increase foraging efficiency, reduce predation risk [26, 27], increase chances of pairing with a familiar partner [28], maintain social status [29], and reduce ectoparasitism [30], all of which facilitate nesting success and offspring survival [21, 31]. In contrast, the reuse of occupied-nests with genetically unrelated young could be a case of the saturation of breeding sites that force breeding pairs to oviposit in an occupied site [32] or of conspecific attraction because the presence of conspecifics may represent the quality of breeding resources [22–24]. Furthermore, for species with parental care, reusing a nest has additional ecological consequences. An adult may raise two cohorts of its own offspring simultaneously when it reuses its own nest [9, 10]. In contrast, when breeding in the nests of others, adults may raise genetically unrelated offspring (if it reproduces and stays) or be a brood-parasite (if it reproduces and leaves). Parental care is energetically expensive, and an investment in genetically unrelated young usually will decrease the ability of an animal to produce its own offspring, thereby reducing its fitness [33].

In this study, we used a Taiwanese frog (*Kurixalus eiffingeri* (Anura: Rhacophoridae)) that breeds in water-filled bamboo stumps as a model animal to study the parentage between overlapping offspring and its ecological consequence on reproductive strategy and nest site selection. Specifically, we used (1) five microsatellite DNA markers to analyze the parentage of adults and tadpoles and (2) paired bamboo cups with and without tadpoles to study the nest choice of frogs and to reveal the possible causes of nest reuse.

Amplectant pairs of *K*. *eiffingeri* lay fertilized eggs above the water line on the inner walls of bamboo stumps or tree holes [34]. It exhibits complex parental care which includes paternal care to eggs and subsequent maternal care to larvae. Male frogs attend nests during the embryonic stages (ca. 10–14 d), and they leave the stumps after embryos have hatched and do not defend the nest site afterwards [35]. Tadpoles live the water pool where they grow and develop until metamorphosis (larval period is about 45–60 d). Tadpoles are obligatorily oophagous and are fed by females that lay unfertilized, trophic eggs directly in the water, in the absence of male frogs [36]. Female frogs visit and feed tadpoles at intervals of about 8 d, and feeding occurs only at night [37]. Females leave the nest after feeding and do not have any other form of care (such as defending a nest site against predators). In the field, we have observed egg-attending males calling and obtaining a new batch of eggs, resulting in overlapping offspring in slightly different developmental stages. Calling to attract females during egg attendance is an alternative reproductive strategy to compensate for the loss of future mating opportunities [38]. In addition, we have also observed that frogs oviposited in tadpole-occupied bamboo stumps, resulting in a coexistence of offspring with different life stages (i.e., embryonic and larval stages) [39, 40]. Upon hatching, newly-hatched tadpoles coexist with larger, older tadpoles from an early-laid egg clutch [39]. Given that the timing of deposition of two egg clutches is separated temporally, we predicted that these breeding patterns (oviposition in egg- or tadpole-occupied stumps) represents different reproductive strategies; consequently, the parentage between the adults and the two cohorts of offspring is expected to be different.

Water pools in bamboo stumps, like other phytotelmata, are ephemeral habitats that are structurally simple and prone to desiccation, but the quality and availability of these reproductive resources vary in time and space [41–44]. Earlier studies on *K*. *eiffingeri* demonstrated severe inter-clutch competition for food between successive cohorts of tadpoles. Larger, older tadpoles from the early-laid clutch outcompete smaller, younger tadpoles from the late-laid clutch and suppress their growth and development [39, 45]. In *K*. *eiffingeri*, the oviposition decisions of frogs can be time- and context-dependent, and some breeding sites can be repeatedly used throughout breeding season, resulting in overlapping cohorts in a pool [40]. However, the parentage between adults and tadpoles is unknown, thus the reproductive strategies cannot be determined. Given that the availability of breeding resources is limited and inter-clutch competition of tadpoles is severe, we predicted that if a stump is reused, it is most likely used by the same male or female frog because breeding sites are hard to locate and the familiarity of a nest site facilitates the reproductive success of the parents.

## Materials and Methods

### Study site

We conducted experiments in the bamboo forests at Chitou in Nantou County, Taiwan. The average monthly temperature is 18 °C, and the total annual rainfall is about 3000 mm [46]. The rainy season is from February to September. Local farmers cut bamboo (*Phyllostachys edulis*) for commercial purposes, and the stumps collect rain water and become suitable breeding sites for *K*. *eiffingeri* [46].

### Field Procedure

#### Survey and sample collection of overlapping clutches

From March—August 2007–2009, we conducted field surveys to collect adults (males and/or females) and overlapping offspring (eggs and tadpoles) for parentage analyses. We first surveyed bamboo forests and located bamboo stumps containing tadpoles. We marked the stumps and monitored them if an egg clutch was found in the inner wall of stumps above the water line. We surveyed stumps every 3–7 d., dependent upon the breeding activity of frogs. Once an egg clutch was found in a tadpole-occupied stump, we collected all eggs and tadpoles about five days later and reared them in the laboratory in order to sample tissues for parentage analyses.

We captured and toe-clipped attending male frogs and preserved the tissue individually in 95% ethanol. Feeding females were more difficult to capture because they return to the nest during the night at an interval of about eight days [37]. Nevertheless, from June 27 to August 31, 2009, we set up funnel traps on the opening of stumps to trap feeding females [37]. If an egg clutch was deposited in a tadpole-occupied stump, we would remove eggs and tadpoles and transport them back to the laboratory about five days later. To continue the trapping procedure, we put ten tadpoles that had been collected from bamboo stumps at least 1–2 km away into the emptied stump. Female *K*. *eiffingeri* do not discriminate kin from non-kin[37], and we could capture them while they were feeding the substitute tadpoles. Upon capture, we toe-clipped female frogs and preserved the tissue individually in 95% ethanol.

In the laboratory, we incubated egg clutches separately on moist substrates until hatching. We reared tadpoles in beakers (ca. 1 L water) and fed them with chicken egg yolk once every 4 days until they reached metamorphosis. Chicken egg yolk is a good substitute for *K*. *eiffingeri* eggs [47]. When tadpoles reached Gosner stage 40, we clipped a distal portion of tadpoles’ tail (i.e., 10% or less of total tail length) and preserved tissues in 95% ethanol for parentage analyses.

#### Nest choice experiment

**Fig 1:**
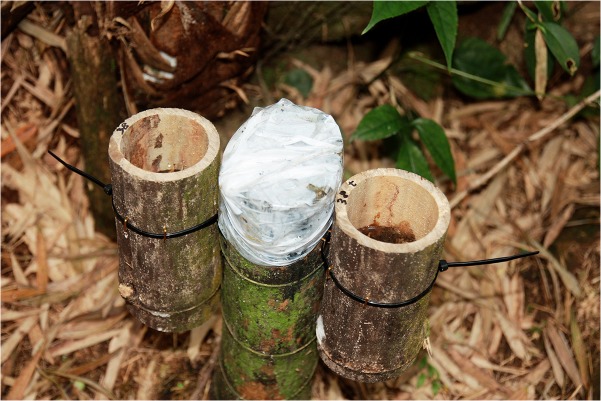
A set-up for a manipulated experiment on nest choice. Paired bamboo cups were attached to a stump which was covered by a plastic sheet.

From April 17 to August 15, 2013, we conducted a manipulated experiment where paired bamboo cups, with and without tadpoles, were set up to investigate nest choice of frogs. First, we collected bamboo trunks which were cut by local farmers to thin the forest and sawed a section of trunk (i.e., an internode) which contains a septum at each end. We then sawed the middle of the internode which resulted in two identical bamboo cups and tied them to a bamboo stump (Fig 1). Bamboo stumps were covered by plastic sheet to prevent frogs from depositing eggs. We randomly designated one cup as the control and the other as the experimental group. A control group contained water only but the experimental group contained water and 5 tadpoles (Gosner stage 28–35). We filled the paired-bamboo cups with 7 cm of water [46]. We drilled small holes in the bamboo wall to prevent a rise of the water level from rainfall and, where necessary, added water to prevent the drop of water level by evaporation [25]. Tadpoles were collected from bamboo stumps at least 1–2 km away. We set up 50–80 paired-bamboo cups each week during the study period, dependent upon the breeding activity of frogs and availability of tadpoles.

We surveyed the paired-bamboo cups every 4 days. Once fertilized eggs were found on the inner wall of a bamboo cup above the water line, we recorded which cup was used and the clutch size. Subsequently, these paired-bamboo cups were no longer used for the rest of our experiment, and we usually set up new paired-bamboo cups in nearby bamboo forests. In each survey, we removed the tadpoles from the bamboo cups, fed them with raw chicken egg yolk in a water bowl, and returned them to bamboo cups after feeding [47]. If tadpoles reached metamorphic stages, Gosner stage 40 or older, we replaced them with younger tadpoles.

### Genetic analyses on the parentage of tadpoles and adults

Detailed methods on parentage analyses were reported by Chen et al [48]. Briefly, we used five polymorphic microsatellite loci (CEd12365, CEd15688, CEd19063, CEd13854, and CEd19091) to conduct parentage analyses [48]. At first, DNA was extracted from tissues, amplified, and genotyped using standard methods [48]. Program Cervus 3.0 [49, 50] was used to calculate the number of alleles, allele frequencies, null allele frequencies and exclusion probabilities for each locus, and the combined exclusion probability. The combined exclusion probability across all loci was 0.987 when the genotype of a parent is known [48]. The likelihood-based, COLONY 2 program [51] was used to analyze genetic relationships between the attending males, feeding females and the offspring in the nests.

Based on the results of the parentage analyses, we deduced the mating pattern of frogs (S1 Table). We defined (1) a synchronous polyandrous female as one which mates with multiple males at the same time [15]; (2) a sequentially polygynous male as one which mates with several different females at different periods of time [38]; (3) a sequential multi-mating event as a series of matings that occur within a short time, such as in a night or within a week. Each mating could be monogamous, polyandrous, or polygynous.

Also, based on the results of parentage analyses, we further assessed whether individuals that breed earlier in a stump would reuse the same stump again. For example, if the early-laid clutch is a result of mating by a male (A) and female (X) and the late-laid clutch by two males (A and B) and a female (Y), then we conclude that the tadpole-occupied stump was reused by the same male (i.e., A) but different females (i.e., X and Y). In contrast, if the early-laid clutch is a result of mating by two males (C and D) and a female (Z) and the late-laid clutch by a male (E) and female (Z), then we conclude that the tadpole-occupied stumps were reused by the different males (i.e., C, D, and E) but same female (i.e., Z).

### Statistical analyses

All statistical analyses of the data were performed with SAS [52]. We used a G test to assess the occurrence of stump use by frogs and proportion of nest reuse among sexes [53]. We used the Wilcoxon rank sum test to compare the clutch size of eggs laid in empty and tadpole-occupied stumps. Unless stated otherwise, we report the mean ± SD of each variable.

### Ethic statement

*Kurixalus eiffingeri* is not an endangered or protected species and thus no specific permission is needed for field studies. Some bamboo forests in Chitou are privately owned, and we had verbal permission from land owners (Mr. Pan Yeong-Sung and Ms Chen Mei-Chi) to conduct observational and field studies (23^o^41’05.4” N 130^o^47’45.4” E and 23^o^41’22.8” N 130^o^47’22.5” E, respectively).

Institutional Animal Care and Use Committee (IACUC) of Tunghai University has specifically approved this study (Approval No. 100–19). We captured and toe-clipped attending males and feeding females and preserved the tissue individually in 95% ethanol for parentage analyses. Because only the most distal segment of the second toe was clipped, the toe-clipping is likely to have a minimal effect on the individual [48, 54]. When tadpoles reached Gosner stage 40, we clipped a distal portion of tadpoles’ tail (i.e., 10% or less of total tail length) [48]. Due to the fast healing of wounds (1–2 days) and sedentary nature of tadpoles, the tail-clipping is likely to have little effect on activity and feeding of tadpoles [38, 48].

## Results

### Field observation and sample collection for parentage analyses

**Fig 2:**
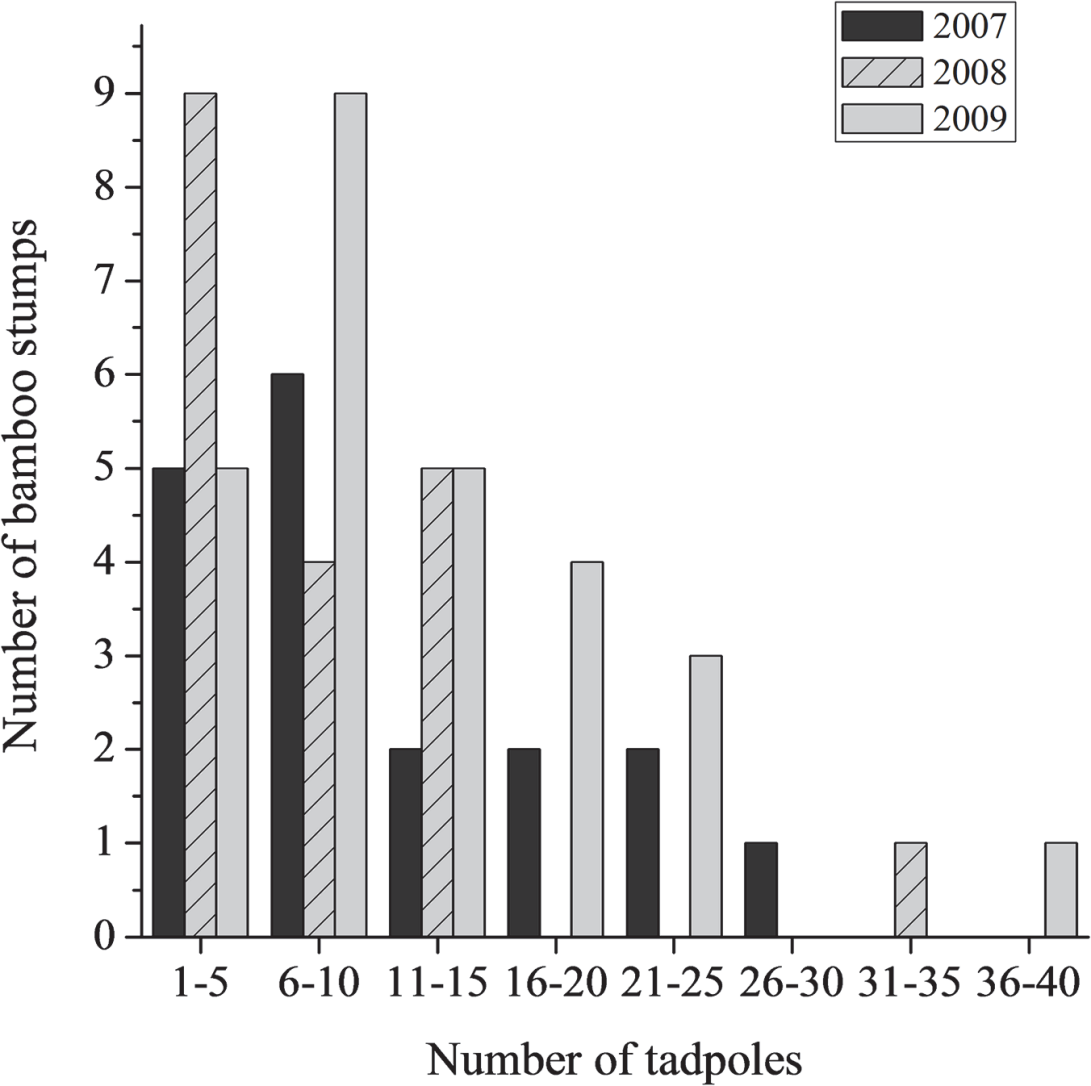
The number of tadpoles found in water pools of bamboo stumps. The number of tadpoles was counted when an egg clutch was deposited above the water line on the inner wall of the stump.

We located 64 bamboo stumps in total that contained eggs and tadpoles simultaneously (18, 19, and 27 stumps in 2007–2009, respectively). The average number of eggs (i.e., late-laid clutch) and tadpoles (i.e., early-laid clutch) found in the stumps was 44.3 ± 24.6 (range: 10–155) and 10.5 ± 7.7 (range: 2–37 tadpoles), respectively (Fig 2).

We selected 24 out of 64 stumps to conduct parentage analyses. The selected stumps consisted of at least one sampled adult (attending male and/or feeding females) and 6 tadpoles each from early- and late-laid clutches (12 tadpoles total). We analyzed a total of 301 and 451 samples from early- and late-laid clutches, respectively.

### Parentage analyses

COLONY deduced eight different types of parentage among overlapping offspring (Table 1). The most common parentage among overlapping offspring was partially the same father and different mothers (*N* = 8), followed by different parents (*N* = 7). The former is a case when a male frog was involved in matings that sired offspring in the early- and late-laid clutches, and at least one of the clutches was either synchronous polyandry or sequential multi-mating events that included other male frogs, which resulted in multiple paternity. In contrast, the latter is a rather simple case where two different pairs of frogs produced the two cohorts of offspring. The remaining types of parentage among overlapping offspring occurred at much lower frequency (Table 1 and S1 Table).

**Table 1:** Parentage analyses of overlapping offspring of three consecutive years using COLONY.

Parentage	2007	2008	2009	Subtotal
Same tale an	0	0	1	1
Same ♂ame tale ly same ♀	0	0	1	1
Same ♂ameetale an ♀	0	2	1	3
Partial same ♂arti ♀	0	0	1	1
Partial same ♂ardifferent ♀	2	2	4	8
Different ♂iffere ♀	0	0	2	2
Different ♂ifferententaly ♀	0	0	1	1
Different ♂iffe ♀	2	2	3	7
	4	6	14	24

Partial same ♂: a male is the genetic father of the two cohorts of offspring while at least one of them is not completely sired by him.

Partial same ♀: a female is the genetic mother of the two cohorts of offspring while at least one of them is not completely produced by her. In other words, at least one of the cohorts may contain offspring from several egg clutches with similar developmental stages which were mistakenly identified as one egg clutch when collected.

Based on results of parentage among overlapping clutches, we found that a total of 14 stumps were reused by the same male frogs but 10 stumps were not, and the proportion of stump use is statistically similar (G test, G = 0.670, *P* = 0.413). On the other hand, a total of 6 stumps were reused by the same female frogs but 18 stumps were not, and the proportion of stump use was statistically different (G test, G = 6.279, *P* = 0.012).

### Nest choice experiment

We conducted 28 surveys, checking a total of 1862 cup pairs from April to August and found that 57 egg clutches were deposited during the study period (Table 2). Most egg clutches were found between May to July when the breeding activity peaked. A total of 34 and 23 egg clutches were laid in control and experimental bamboo cups, respectively, and the egg placement was independent from cup treatment (G test, G = 2.136, *P* = 0.144). During this three month period (May to July), 40, 31, and 52% of egg clutches were laid in the experimental cups in the respective months. Clutch size of eggs laid in control cups (45.3 + 18.4 eggs, *N* = 24) was not different from that of experimental cups (42.8 + 19.4 eggs. *N* = 17; Wilcoxon rank sum test, W = 229.5, *P* = 0.508).

**Table 2:** Survey effort and frequency of egg clutches laid in empty or tadpole-occupied cups.

	Number of surveys per month	Number of cup checked per survey	Total number of cups checked per month	Frequency of egg clutches laid in empty cups	Frequency of egg clutches laid in tadpole-occupied cups	Subtotal egg clutches laid
April	2	34–56	90	1	0	1
May	6	47–77	364	6	4	10
June	7	71–78	526	15	7	22
July	9	62–77	608	11	12	23
August	4	65–70	274	1	0	1
			1862	34	23	57

## Discussion

### Multiple parentage of overlapping offspring

Overlapping offspring is a reproductive situation with ecological and evolutionary implications. Attending male fishes [55, 56] and frogs including *K*. *eiffingeri* [5, 6, 8, 38, 57] obtain additional egg clutches in a nest, resulting in overlapping egg clutches. The occurrence of overlapping egg clutches in a nest has been proposed as a reproductive strategy used by attending males to compensate for loss of mating opportunities while attending eggs [5, 8, 58–60]. In *K*. *eiffingeri*, Cheng et al. [38] used microsatellite DNA markers to analyze parentage and showed that most overlapping egg clutches in a stump were a result of sequential breeding (or polygyny) of the attending male, providing direct evidence to support earlier predictions that attending anuran males can sire and care for multiple egg clutches in a single egg deposition site.

In this study, *K*. *eiffingeri* oviposited in tadpole-occupied stumps, resulting in overlapping offspring with different life stages; molecular and field evidence strongly suggest that the overlapping offspring in this study is not a continuum of overlapping egg clutches reported earlier [38] and represents a different breeding phenomenon. Two lines of evidence support our contention: first, only 50% of overlapping offspring in this study were sired (completely or partially) by the same male frog compared to 93% of overlapping egg clutches in Cheng et al. [38]; second, the earlier field evidence indicates that the prevalence of overlapping offspring with different life stages was about 36% [40] compared to only 5% of overlapping egg clutches in Cheng et al. [38]. In fact, overlapping offspring of different life stages in this study is more similar to the breeding strategy employed by pigeons [9, 10, 12] and fishes [3].

The casual mechanism(s) of the varied patterns of parentage in overlapping offspring in this study are yet to be clarified and are expected to be more complicated than that of overlapping egg clutches reported earlier. We speculate that the varied parentage patterns can at least be associated with two ecological factors: a lack of nest defense and limited breeding resources. *Kurixalus eiffingeri* has a unique “sequential” form of parental care: male frogs guard eggs during embryonic period, and female frogs feed tadpoles alone during larval period; however, there is no evidence of nest or mate defense. Nest defense is a common parental behavior among insects [61], fishes [62], salamanders [63–65], frogs [66, 67], and birds [68–70] and functions to protect valuable resources inside (e.g., mates, offspring, food, even the nest site itself [61]). In birds, the increased frequency of extra-pair mating or paternity has been associated with a lack of territorial or mate-guarding behavior [71–74]. This may also be true in *K*. *eiffingeri* in that a lack of mate and nest defense opens possibilities for a male or female frog to mate with others and utilize the tadpole-occupied nests to breed again. Furthermore, the quality and availability of water pools in stumps vary in time and space [40]. As the reproductive season progresses, more and more of the arboreal pools may already have been used for breeding by other individuals [40]. The competition for stumps (empty or tadpole-occupied) for breeding is heavy, which inevitably results in the reuse of tadpole-occupied stumps. This likely leads to a diverse pattern of parentage in the overlapping offspring.

The lack of nest defense during the larval period by both parents comes as a surprise in light of the importance of the nest site and brood inside. Hom et al. [75] proposed a dynamic optimization model to predict the fitness consequences of nest defense, and one of the predictions is that nest defense would be minimal or lacking for species with small body size. *Kurixalus eiffingeri* is a small-size frog (snout-vent length 2–3 cm) which is probably ineffective in protecting tadpoles from snake predation, the primary cause of tadpole mortality [76]. In addition, the lack of nest defense by males during the larval period is probably because the benefit of seeking other stumps to breed in right away outweighs the costs of breeding in the same nest [40]. In females, a lack of nest defense may also relate to the feeding scheme. Each *K*. *eiffingeri* tadpole consumes nearly 80 eggs to support its growth and development until metamorphosis [46], thus, egg provisioning to tadpoles is a time consuming process. As a result, female frogs are forced to spend a large amount of time away from the nest foraging and producing trophic eggs to meet the energetic demands of their tadpoles.

### Oviposition strategies when reusing a nest

Our findings that 58 and 25% of *K*. *eiffingeri* males and females respectively reused the same stump to breed partly agreed with our prediction. Earlier studies have reported site fidelity in stream-dwelling frogs which probably are able to obtain sufficient ecological necessities such that moving away from a site is not required [77–79]. Results from studies in birds have demonstrated that nest site fidelity facilitates nesting success and offspring survival [21, 31, 80]. Accordingly, these beneficial effects should apply to *K*. *eiffingeri* that reuses a previously-used stump. However, females reused nests at a much lower rate, suggesting a sexual difference in nest reuse. Earlier studies showed that the degree of nest-site fidelity can be influenced by previous experience [81], nest competition [82], age [31, 80, 83], site quality [24], and predation [26]. In this study, the lower rate of reusing the same nest by females can probably be explained by the high cost of tadpole feeding. Tadpoles are obligatorily oophagous, and each tadpole consumes about 80 trophic eggs during larval period (45–60 d) [46]. The feeding cost for a female to raise about 20 tadpoles (i.e., average tadpole number per stump) is extremely high [84] preventing her from breeding again while caring for the tadpoles. However, when tadpoles begin to metamorphose and leave the stumps, female frogs may breed again when tadpoles are few [40], and the feeding cost is presumably lower.

On the other hand, results of parentage analyses showed that about 42 and 75% of males and females respectively bred in tadpole-occupied stumps where tadpoles were genetically unrelated, which leads to the following question: why did frogs breed in the tadpole-occupied stumps of others? Lin, Lehtinen and Kam [40] reported that reuse of nests by *K*. *eiffingeri* occurred more commonly in the second half of breeding season. One explanation is that amplectant pairs may be forced to lay eggs in tadpole-occupied nests when most suitable breeding habitats are already used later in the breeding season (i.e., the saturated habitat hypothesis) [32]. These tadpole-occupied stumps are less favorable nests because larger tadpoles from early clutches outcompete smaller tadpoles from late clutches for trophic eggs that may result in tadpole starvation, retarded growth, and mortality of the latter [39, 45]. In theory, amplectant pairs would avoid tadpole-occupied stumps to minimize the detrimental impact of residing tadpoles to their offspring. However, in the nest choice experiment, when we presented identical stumps, without or with tadpoles, 40% of frogs still chose tadpole-occupied stumps, suggesting that the habitat saturation hypothesis does not fully explain why frogs used the tadpole-occupied stumps. One explanation is that females are unable to detect the tadpoles in the stumps, and the chance of stump use is random. This possibility, however, is unlikely because adults are selective in oviposition sites [46, 85], and females purposely deposited egg clutches in stumps with fewer tadpoles [40]. The alternative explanation for the re-use of stumps is that even though the quality of tadpole-occupied stumps is discounted due to inter-clutch tadpole competition, they are still as good as, if not better than, the remaining unoccupied stumps [40].

What are the possible benefits of reusing tadpole-occupied stumps? First, the presence of early-clutch tadpoles may serve as a cue for the high quality of the stumps, such as the availability and persistence of the water resource, which is particularly critical for offspring living in container habitats like tree holes and stumps which are prone to desiccation [24, 41, 43]. Similar findings of conspecific attraction have been reported in vertebrates and invertebrates [22–24, 53]. When two cohorts of offspring with different developmental stages coexist, the negative effect of early-clutch tadpoles on late-clutch tadpoles is unavoidable[45]. However, *K*. *eiffingeri* reuse stumps when residing tadpoles are usually at the middle or late developmental stages and low in number [39, 40], the negative effects of early-clutch tadpoles would be somewhat alleviated because they would soon metamorphose, minimizing inter-clutch competition [45]. Second, the coexistence of two cohorts of tadpoles reduces the probability of smaller tadpoles (i.e., late-laid clutches) being eaten not only because of the attack abatement effect but also because they are less conspicuous due to small size when compared to larger tadpoles (i.e., early-laid clutch) [32]. Third, coexisting tadpoles in stumps could potentially be fed by two females which reduces the probability of catastrophic nest mortality. Earlier studies on *K*. *eiffingeri* have reported nest failure (ca.30%) which is due to nest abandonment by females and/or death of female frogs, mostly by snake predation [45, 84, 86]. Since females cannot discriminate kin from non-kin [37], if a nest is used by two females (i.e., multiple feeders effect), the tadpoles would still be fed if one of the mothers deserts the nest or is eaten. A similar scenario was reported by Harris et al. [87] in that joint nests by *H*. *scutatum* were deserted less often compared to solitary nests, and the probability of catastrophic mortality was accordingly reduced.

## Conclusions

In conclusion, integrating parentage into the discussion of nest site selection can lead to new insights into the reproductive strategies and sexual selection of animals. This is particularly true in the studies of reproductive behavior of externally fertilizing animals such as anuran amphibians and fishes because many species have complex reproductive behavior that cannot easily be detected in the field [13, 16, 38, 88, 89]. In this study, *K*. *eiffingeri* may oviposit in egg- or tadpole-occupied stumps, both resulting in overlapping offspring. However, parentage data and field evidence suggest that oviposition in stumps occupied by either eggs or tadpoles should be seen as two different reproductive phenomena, most likely with different causal mechanisms. Oviposition in egg-occupied stumps is mostly initiated by attending males which probably attempt to compensate for the loss of reproductive opportunities while attending eggs. On the other hand, based on the results of the nest choice experiment in the field, the occurrence of oviposition in tadpole-occupied stumps cannot be fully explained by the habitat saturation hypothesis. We propose several hypotheses to explain the potential adaptive values of overlapping offspring. However, additional studies are necessary to fully understand the patterns revealed by this study.

## Supporting Information

S1 Table Summaries of stump use, clutch size development stage, sample size used, and parentage of overlapping offspring deduced from the COLONY program. (PDF)
