# Correction: Parentage of Overlapping Offspring of an Arboreal-Breeding Frog with No Nest Defense: Implications for Nest Site Selection and Reproductive Strategy

**DOI:** 10.1371/journal.pone.0128483

**Published:** 2015-05-11

**Authors:** Wan-Ping Tung, Yi-Huey Chen, Wei-Chun Cheng, Ming-Feng Chuang, Wan-Tso Hsu, Yeong-Choy Kam, Richard M. Lehtinen

There are errors in Table 1. Please see the correct Table 1 here.

**Table 1:** Parentage analyses of overlapping offspring of three consecutive years using COLONY.

Parentage	2007	2008	2009	Subtotal
Same ♂ and ♀	0	0	1	1
Same ♂, partially same ♀	0	0	1	1
Same ♂, different ♀	0	2	1	3
Partial same ♂ and ♀	0	0	1	1
Partial same ♂, different ♀	2	2	4	8
Different ♂, same ♀	0	0	2	2
Different ♂, partial same ♀	0	0	1	1
Different ♂ and ♀	2	2	3	7
	4	6	14	24

Partial same ♂: a male is the genetic father of the two cohorts of offspring while at least one of them is not completely sired by him.

Partial same ♀: a female is the genetic mother of the two cohorts of offspring while at least one of them is not completely produced by her. In other words, at least one of the cohorts may contain offspring from several egg clutches with similar developmental stages which were mistakenly identified as one egg clutch when collected.
