# Supplementary material for: Parentage of Overlapping Offspring of an Arboreal-Breeding Frog with No Nest Defense: Implications for Nest Site Selection and Reproductive Strategy
Source: PLoS One. 2015 Apr 2;10(4):e0123221. doi: 10.1371/journal.pone.0123221 (PMC4383374; doi:10.1371/journal.pone.0123221)
Supplement: S1 Table — (PDF) [file pone.0123221.s001.pdf]

S1 Table. Stump information. This table is a summary of stump use, clutch size development stage, sample size used, and parentage of overlapping offspring deduced from the COLONY program.

Stump Number[1]	Date of collection	Tadpole number of early-laid clutch (1) and egg number of late-laid clutch (2)	Development (Gosner stage)[2]	Number of analyzed samples[3]	Mating pattern (# sired offspring)[4]	Result of parentage analyses
I04-1	2007/5/5	1=12	>26	7	♂1♀2(5)	**Different parents**
					♂2♀3(2)	
		2=29	-	23	♂A♀1(23)	
I20	2007/5/26	1=10	>26	10	♂1,2♀1(8)	**Partially the same father but different mothers**
					♂2♀2(2)	
		2=52	20	42	♂A,2♀3(36)	
					♂A,2♀4(6)	
N27	2007/5/27	1=21	26-29	12	♂1♀2(12)	**Different parents**
		2=59	21	34	♂A♀1(34)	
B12	2007/6/22	1=16	>26	13	♂1♀2(13)	**Partially the same father but different mothers**
		2=25	-	11	♂A,1♀1(11)	

I43	2008/5/2	1=12	27-29	9	♂A,1♀2(9)	**Same father but different mothers**
		2=28	20	20	♂A,1♀1(20)	
J31	2008/6/24	1=14	26-42	12	♂1,2,3♀1(12)	**Different parents**
		2=10	24	8	♂A♀2(8)	
H14	2008/7/2	1=13	26-37	11	♂A♀1(8)<br>♂A♀2(3)	**Same father but different mothers**
		2=32	-	20	♂A♀3(19)<br>♂A♀4(1)	
G39	2008/7/2	1=32	30-42	20	♂A,1♀1(20)	**Partially the same father but different mothers**
		2=68	-	20	♂A♀2(20)	
B70	2008/7/2	1=15	27-42	15	♂A,1♀1(12)<br>♂1♀2(3)	**Partially the same father but different mothers**
		2=44	-	20	♂A♀3(20)	
H01	2008/7/22	1=12	26-37	10	♂1♀1(4)<br>♂1♀2(6)	**Different parents**
		2=38	-	17	♂A♀3(17)	

Y07-B	2009/5/17	1=14	>26	14	♂A♀F1(14)	**same parent**
		2=21	-	18	♂A♀F1(18)	
Y50	2009/6/6	1=19	26-42	19	♂A♀2(19)	**Same father but different mothers**
		2=18	11	13	♂A♀1(13)	
Y74	2009/6/13	1=17	>26	11	♂1♀1(11)	**Different fathers but the same mother**
		2=33	22	17	♂A♀1(17)	
Y42	2009/6/27	1=25	>26	14	♂1♀F(13) ♂2♀1(1)	**Different parents**
		2=59	15-16	17	♂3♀2(17)	
Y12	2009/6/28	1=12	28-29	10	♂A.1♀2(10)	**Partially the same father but different mothers**
		2=60	10	18	♂A♀1(18)	
X01	2009/6/28	1=11	26-27	11	♂1♀F1(8) ♂2♀1(3)	**Partially the same father but different mothers**
		2=88	10	19	♂A,2,3♀F2(19)	
Y95	2009/7/1	1=37	>26	20	♂A,1♀2(20)	**Partially the same father but different mothers**
		2=42	20	20	♂A♀1(20)	
Z22	2009/7/9	1=23	26-42	17	♂1,2♀F(17)	**Different parents**
		2=32	-	17	♂A♀1(17)	

Y133	2009/7/10	1=6	27-30	6	♂A♀1(2)<br>♂A,1♀2(4)	**Partially the same father and mothers**
		2=95	17	18	♂A,1♀2(11)<br>♂A,1♀F(7)	
A01	2009/7/12	1=23	26 -42	20	♂A,1♀1(14)<br>♂2♀F(6)	**Partially the same father but different mothers**
		2=33	20	20	♂2♀2(20)	
Y98	2009/7/21	1=17	>26	10	♂1♀F(10)	**Different father but same mothers**
		2=45	10	19	♂2♀F(19)	
Y146	2009/7/25	1=19	>26	15	♂A♀F1(7)<br>♂1♀1(4)<br>♂1♀F2(4)	**Partially the same father and mother**
		2=66	-	16	♂A♀F2(16)	
A02	2009/7/29	1=10	>26	9	♂1♀1(4)<br>♂2♀2(5)	**Different parents**
		2=34	15-16	8	♂A♀F(8)	
Y39	2009/7/29	1=6	>26	6	♂1♀1(4)<br>♂1♀F(2)	**Different fathers but partially the same mother**
		2=42	-	16	♂A♀F(16)	

[1]: Stumps were labeled with an alphabetical number (i.e., the location of bamboo forest) followed by stump number in each bamboo forest.

[2]: "-"represent developmental stage of eggs was not recorded at the time of collection

[3]: For the early-laid offspring, we analyzed all of them, if possible, because they were usually less in number. For the late-laid offspring, we analyzed a maximum of 20 tadpoles except in 2007 when we analyzed all tadpoles, if possible..

[4]: ♂A and ♀F represent attending male and feeding female, respectively, whereas ♂x and ♀x represent non-captured, unmarked male x and female x, respectively. For example,♀F1 and ♀F2 represent feeding female 1 and 2; ♂A,1♀2(12) represent 12 offspring were sired by an attending and a non-captured, unmarked male 1 who mated with a non-captured, unmarked female 2. This mating pattern is a case of synchronous polyandry.
